# Association between Antidepressants and Dementia Risk in Older Adults with Depression: A Systematic Review and Meta-Analysis

**DOI:** 10.3390/jcm12196342

**Published:** 2023-10-03

**Authors:** Grace Hsin-Min Wang, Piaopiao Li, Yehua Wang, Jingchuan Guo, Debbie L. Wilson, Wei-Hsuan Lo-Ciganic

**Affiliations:** 1 Department of Pharmaceutical Outcomes and Policy, College of Pharmacy, University of Florida, Gainesville, FL 32610, USA; hsinminwang@ufl.edu (G.H.-M.W.); piaopiao.li@ufl.edu (P.L.); yehua.wang@ufl.edu (Y.W.); guoj1@ufl.edu (J.G.); debbie.wilson@ufl.edu (D.L.W.); 2 Center for Drug Evaluation and Safety, College of Pharmacy, University of Florida, Gainesville, FL 32610, USA

**Keywords:** depression, antidepressant, dementia, older adults, systemic review, meta-analysis

## Abstract

Depression, commonly treated with antidepressants, is associated with an increased risk of dementia, especially in older adults. However, the association between antidepressant use and dementia risk is unclear. We searched for randomized controlled trials and observational studies from PubMed, Embase, and Cochrane on 1 February 2022, restricting to full texts in English. Since dementia is a chronic disease requiring a long induction time, we restricted studies with ≥1 year follow-up. We extracted the relative risk (RR) adjusted for the most variables from each study and evaluated the heterogeneity using I square (I^2^). The protocol was registered in the PROSPERO International Register of Systematic Reviews (CRD42022338038). We included six articles in the systematic review, of which the sample size ranged from 716 to 141,740, and the median length of follow-up was 5 years. The pooled RR was 1.21 (95% CI = 1.12–1.29) with an I^2^ of 71%. Our findings suggest that antidepressant use was associated with an increased risk of dementia in older adults with depression, yet moderate to high heterogeneity existed across studies. Future work accounting for the depression progression is needed to differentiate the effect of depression and antidepressants on dementia risk.

## 1. Introduction

Dementia, a neurodegenerative disorder affecting 14% of adults aged 70 years or older [1], is characterized by a significant decline in memory, thinking, and the ability to perform daily activities [2]. Patients’ cognitive performance continues to worsen over time, leading to a loss of daily autonomy in the final stage of dementia [3]. The global disease burden (GBD) of dementia has been increasing dramatically as life expectancy increases [3], with an estimated total lifetime cost of care for a patient with dementia of USD 412,936 in 2022 [4]. It is estimated that by 2050, around 153 million people worldwide will have dementia [5]. Depression, a mood disorder that causes a persistent feeling of sadness and loss of interest [6], is also common, which occurs in one in five individuals throughout their lifetime [7] and dramatically increased after the COVID-19 pandemic. It is estimated that 53·2 million additional cases of major depressive disorder were due to the COVID-19 pandemic globally [8]. Additionally, depression is among the leading causes of GBD, especially among people aged 15–49 years [9]. Depression and dementia are interrelated in complicated ways. First, depression can impair cognitive function and result in “pseudodementia” [10]. Second, individuals with depression occurring prior to the age of 60 years have been associated with up to two-fold risk of developing subsequent dementia compared with those without depression before the age of 60 years [11]. Third, depression can be a prodromal symptom or an early manifestation of dementia [12]. Finally, patients with dementia can also develop depressive symptoms (i.e., neuropsychiatric symptoms of dementia) [13], of which the occurrence ranges from 30% to 50% [14]. Therefore, controlling patients’ depressive symptoms may be beneficial to their cognitive function, thereby decreasing the risk of dementia.

Pharmacological therapies are primary interventions for depression [15]. Antidepressants are categorized into several classes based on different pharmacological mechanisms of action, with varied safety profiles [16]. For example, tricyclic antidepressants (TCAs, e.g., imipramine) may raise safety concerns in older adults due to their strong anticholinergic effect, which is associated with increased intraocular pressure, urinary retention, dry mouth, postural hypotension, and falls [16]. In addition, anticholinergic drugs can block muscarinic acetylcholine receptors in the nervous system that are critical to the brain areas involved in cognitive function and in the pathophysiology of Alzheimer’s disease (the most common subtype of dementia), thereby increasing the risk of dementia [17]. The 2019 AGS Beers Criteria also recommended against the use of drugs with strong anticholinergic properties (i.e., amitriptyline, amoxapine, clomipramine, desipramine, doxepin, imipramine, nortriptyline, paroxetine, protriptyline, and trimipramine) among older adults with dementia or cognitive impairment [18]. In contrast, selective serotonin reuptake inhibitors (SSRIs, e.g., fluoxetine), selective norepinephrine reuptake inhibitors (SNRIs, e.g., venlafaxine), trazodone, bupropion, mirtazapine, and moclobemide are considered relatively safer in older adults because of fewer anticholinergic effects compared with TCAs [19].

Randomized controlled trials (RCTs) have reported cognitive improvement as a therapeutic benefit of SSRIs [20,21,22,23,24,25] and SNRIs [26]. However, these RCTs followed patients for a relatively short period (i.e., 4 weeks to 1 year), which may not have been long enough for dementia to develop. In fact, a recent prospective cohort study found that antidepressants, especially those categorized as potentially inappropriate medications (PIMs, i.e., imipramine, clomipramine, trimipramine, amitriptyline, doxepin, maprotiline, fluoxetine, tranylcypromine), were associated with an increased risk of dementia over 12 years of follow up [27], which was contrary to the results from RCTs. 

To our knowledge, existing reviews examining the effect of antidepressants on cognitive function only included studies conducted prior to 2018, which may not cover the most recent evidence. Additionally, most did not consider the length of follow-up to account for a long induction time since dementia takes a long time to develop. A meta-analysis of observational studies following patients for 3 to 11 years (median: 6 years) suggested that antidepressant use was associated with an increased risk of dementia compared with no use in elderly patients [3]. However, it was not restricted to patients with depression, resulting in a high heterogeneity (I^2^ = 99%) and thus limiting the reliability of the findings. Thus, we aimed to conduct a systematic review and meta-analysis including the most up-to-date evidence to examine the association between antidepressant use and risk of dementia over a follow-up period of ≥1 year in older adults with depression. Different classes of antidepressants may also affect the association with cognitive function due to their mechanism of action. Thus, we also investigated the effect by grouping patients’ class of antidepressants (i.e., TCA and SSRI since they are the most commonly used classes).

## 2. Materials and Methods

This systematic review and meta-analysis was conducted following the Preferred Reporting Items for Systematic Reviews and Meta-analyses (PRISMA) checklist [28], as provided in Table S1. We prospectively registered the protocol in the PROSPERO International Register of Systematic Reviews (CRD42022338038) [29].

### 2.1. Data Sources and Search Strategies

We searched for articles from PubMed, Embase, and Cochrane on 1 February 2022, restricting the search to articles written in English. Briefly, we searched for (aged OR elderly OR their synonyms) AND (depression OR its synonyms) AND (antidepressant OR its synonyms) AND (dementia OR cognitive impairment OR their synonyms). For example, our keywords for Embase were [(aged OR elderly OR senior OR senium):ti,ab] AND [(depress* OR melancholia*):ti,ab] AND {[(antidepress* OR ((adrenaline OR amine OR monoamine OR monoamino OR mao OR monoaminoxidase) near/3 “oxidase inhibit”) OR ((tyraminase OR “tyramine oxidase”) near/3 inhibit) OR ((noradrenalin OR noradrenaline OR noradrenaline OR norepinephrine) near/3 “reuptake inhibitor”) OR SNRI OR ((“serotonin specific” OR “selective serotonin” OR serotonin OR norepinephrine) near/3 “reuptake inhibitor”) OR SSRI OR tetracyclic near/3 antidepressant OR tricyclic near/3 antidepressant):ti,ab] OR [“monoamine oxidase inhibitor”/exp OR “noradrenalin uptake inhibitor”/exp OR “serotonin uptake inhibitor”/exp OR “tetracyclic antidepressant agent”/exp OR “tricyclic antidepressant agent”/exp]} AND {[((cogniti* AND (deficit OR defect OR disability OR disorder OR dysfunction OR impairment OR decline)) OR dementi* OR amnestic OR amentia):ti,ab] OR [“Cognitive defect”/exp OR “Dementia”/exp]}. The search details for the other databases are listed in Table S2, which were finalized after consultation with a research librarian. After gathering all the potential articles, we imported them into EndNote^®^ X9 and removed the duplicates. 

### 2.2. Article Selection

Three reviewers were involved in the article selection process (GW, PL, and YW), and the results were tracked using an Excel spreadsheet. First, two reviewers independently screened the titles and abstracts of all potential articles. We included reviews, RCTs, and observational studies, such as cohort studies and case-control studies that evaluated the association between antidepressant use and the risk of dementia in older adults, with a follow-up period of ≥1 year. Letters to the editor, editorials, case reports, and case series were excluded. Then, we retrieved the full texts of the included articles, and two reviewers independently reviewed them based on the selection criteria. From reviews, we extracted individual studies relevant to our research question and included them in the full-text assessment; however, reviews were not included in the meta-analysis. Any disagreement throughout the study selection process was resolved by the third reviewer.

### 2.3. Data Extraction

From the included studies, we collected the name of the first author, year of publication, study design (e.g., RCT/cohort study/case-control study), study location, population (i.e., sample size, age, indication for antidepressant use, race and ethnicity), details of antidepressant use (i.e., class, dosage, duration), dementia outcome measures, length of the follow-up period, and main findings of the study with crude and adjusted odds ratio (OR), risk ratios (RRs), and hazard ratios (HRs) as well as 95% confidence intervals (CIs). If multiple classes of antidepressants were discussed in an article, we identified the result for each specific class during categorization. All the above items were documented in an Excel spreadsheet.

### 2.4. Risk of Bias Assessment

We used the latest version of the Cochrane risk-of-bias tool (RoB2) to evaluate the methodological quality of the included RCTs [30]. RoB2 assesses the risk of bias due to randomization, deviations from intended interventions, missing data, outcome measurements, and selection of reported results. Each domain was assigned a bias grade of “low”, “high”, or “some concerns” based on our reviewers’ (GW, PL, and YW) overall risk-of-bias judgement. For observational studies, we used the Strengthening the Reporting of Observational Studies in Epidemiology-Modified (STROBE-M) checklist to evaluate their quality [31]. STROBE-M consists of 22 items related to all sections of a manuscript, including title, abstract, introduction, methods, results, discussion, and conclusion. In addition, STROBE-M was designed to allow for scoring. The fulfillment of each item contributes from 1 to 4 to the STROBE-M score. We obtained the STROBE-M adherence score by dividing the total score by the maximum possible STROBE-M score for a specific study design (cohort = 84, case-control = 83, and cross-sectional = 77). Finally, the quality of the observation study was categorized as “excellent”, “good”, “fair”, or “poor” based on the adherence to STROBE-M (i.e., ≥0.85, 0.70 to <0.85, 0.50 to <0.70, and <0.50, respectively) [31]. The results were tracked using an Excel spreadsheet. To check publication bias, a funnel plot was plotted using Cochrane ReviewManager (RevMan) version 5.4.1.

### 2.5. Data Analysis

Our outcome of interest was dementia, and the exposure of interest included all types of antidepressants. From the identified studies, we restricted the analysis to articles including the information of OR/RR/HR with 95% CI and using non-antidepressant users as the comparator. HR is broadly equivalent to RR [32], and OR provides a reasonable approximation of RR in case-control studies and cohort studies in which the outcome occurs in less than 10% of the unexposed population [33]. We therefore approximated RR with the OR/RR/HR values adjusted for the most covariates from the included articles and pooled them using RevMan. Crude estimates were extracted if no adjusted estimates were available. We reported the pooled RR along with the 95% CI. An increased risk of dementia is indicated if the lower limit of pooled RR is greater than 1; on the contrary, if the upper limit of pooled RR is less than 1, then a decreased risk of dementia is suggested. When an article reported results for different antidepressant classes or different antidepressant doses separately, we used a fixed-effects model to pool results within the same article to obtain the risk of dementia associated with overall antidepressant use, given that the heterogeneity in the same study may not be too high. When pooling results between articles, we used a random-effects model and reported the I square (I^2^) to assess the extent of heterogeneity [34,35], considering the likelihood of heterogeneity between different articles. The empirical thresholds of I^2^ were 25%, 50%, and 75%, indicating low, moderate, and high heterogeneity, respectively. We considered I^2^ of 75% or above as an a priori indication of considerable heterogeneity [34,35]. We also stratified the results by different classes of antidepressants (i.e., SSRI, TCA) because the mechanism of antidepressants may have an impact on cognitive function.

## 3. Results

### 3.1. Literature Search

**Figure 1:**
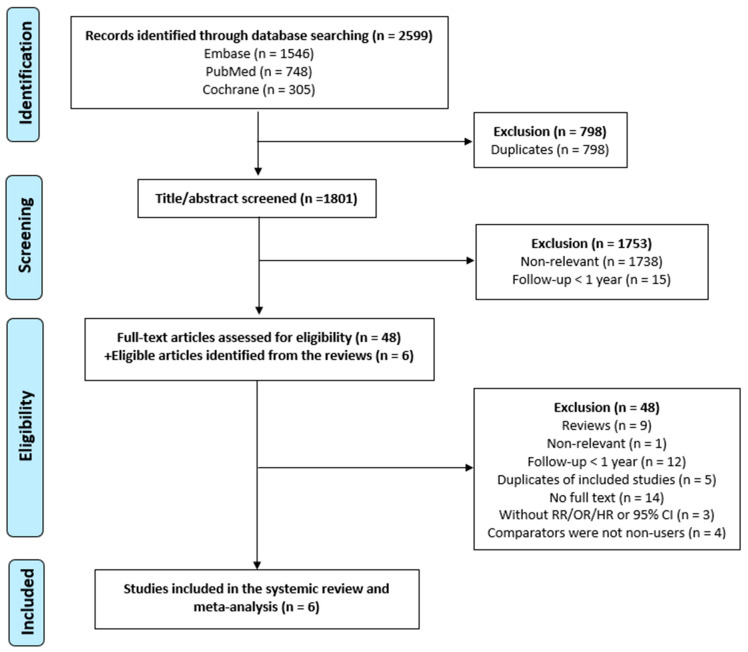
Flowchart showing the study selection process. In total, 2599 articles were identified using the aforementioned search strategies from the three databases (Embase *n* = 1546, PubMed *n* = 748, Cochrane *n* = 305). After excluding 798 duplicates, 1801 studies were screened for the title and abstract. We then excluded 9 reviews, 1 study with irrelevant contents, 12 articles with a follow-up period of less than 1 year, 5 duplicates of included studies, 14 articles without full text, 3 articles without RR/OR/HR or 95% CI reported, and 4 articles not using non-users as the comparator. Eventually, this systematic review and meta-analysis included 6 studies.

We identified 2599 articles from the three databases (Embase *n* = 1546, *n* = PubMed 748, *n* = Cochrane 305) using the search strategies. After excluding 798 duplicates, 1801 studies were screened for the title and abstract. We excluded 1738 articles that were not related to our research question and 15 articles with a follow-up period of less than 1 year. In the full-text assessment, 48 studies were evaluated, and six eligible studies were added from the reviews. We then excluded nine reviews, one study with irrelevant content, 12 articles with a follow-up period of less than 1 year, five duplicates of included studies, 14 articles without full text, three articles without RR/OR/HR or 95% CI reported, and four articles not using non-users as the comparator. Eventually, this systematic review and meta-analysis included six studies. A flowchart showing the study selection process is shown in Figure 1.

### 3.2. Study Characteristics

Of the six included studies, one was an RCT, three were retrospective cohort studies, and two were case-control studies. The RCT was of low concern, and the quality of the observational studies was generally good (*n* = 2) and excellent (*n* = 3) with an average STROBE-M of 0.86 (i.e., excellent quality). Half of the included studies were conducted in the US, two were conducted in Taiwan, and one was conducted in the UK. The majority of the studies included both sexes, except one study (Goveas et al., 2012 [36]) that consisted of postmenopausal women only. The mean age ranged from 71 to 80 years, and the sample size ranged from 716 to 141,740. The length of the follow-up period ranged from 2.7 to 8 years across studies (median: 5 years). A summary and details of patient characteristics are provided in Table 1 and Table S3, respectively.

### 3.3. Summary of the Included Studies

The included studies were summarized and organized by their publication year and ordered alphabetically by the first author’s last name.

Goveas et al., 2012 [36] was an RCT of low concern about the quality conducted in the US. The study population included 6998 postmenopausal women aged 65–79 years who were with depressive symptoms and without dementia (mean age: 71.0 ± 3.9 years, 91% White). The exposure of interest was all types of antidepressants compared to no use. The mean follow-up was 7.6 years. The HR was adjusted for demographic characteristics (e.g., race and ethnicity), socioeconomic status (e.g., education), comorbidities (e.g., diabetes), comedications (e.g., statins), baseline depressive symptoms, physical activity, body mass index, and baseline Modified Mini-Mental State Examination score (i.e., a scale used to indicate cognitive status). The adjusted HR was 1.55 (95% CI = 1.09–2.20) for all antidepressants, 1.50 (95% CI = 0.89–2.53) for SSRI, and 1.75 (95% CI = 1.05–2.91) for TCA, compared with no use.

Chatterjee et al., 2015 [37] was a nested case-control study of good quality (STROBE-M: 0.77) conducted in the US. The study population included 141,740 nursing home older adults with depression and without dementia (mean age: 80.4 ± 7.4 years, 81% female, 86% White). The exposure of interest included drugs with a moderate to high anticholinergic burden (Anticholinergic Drug Scale level of 2 to 3) compared to no use. Antidepressants fulfilling such criteria included amitriptyline, clomipramine, doxepin, desipramine, imipramine, nortriptyline, paroxetine, protriptyline, and trimipramine. The mean follow-up was 3 years. The OR was adjusted for demographic characteristics (e.g., race and ethnicity), comorbidities (e.g., diabetes), comedications (e.g., statins), and duration of depression. The adjusted OR was 1.26 (95% CI = 1.22–1.29) for antidepressant users compared with no use.

Lee et al., 2016 [38] was a case-control study of excellent quality (STROBE-M: 0.92) conducted in Taiwan. The study population included 10,626 patients aged 40 years or older with major depression (mean age: 76.0 ± 8.8 years, 67% female). The exposure of interest included TCAs, SSRIs, monoamine oxidase inhibitors (MAOIs), heterocyclic antidepressants, and other antidepressants compared to no use. The mean follow-up was 5 years. The OR was adjusted for age, sex, other antidepressant use, and comorbidities (e.g., diabetes). The adjusted OR was 0.24 (95% CI = 0.22–0.27) for TCA, 2.48 (95% CI = 2.27–2.71) for SSRI, 1.86 (95% CI = 1.47–2.36) for MAOI, 1.44 (95% CI = 1.32–1.57) for heterocyclic antidepressants, and 2.05 (95% CI = 1.85–2.27) for other antidepressants, compared with no use.

Han et al., 2020 [39] was a retrospective cohort study of good quality (STROBE-M: 0.82) conducted in the US. The study population included 716 older cognitively normal adults with depression (mean age: 71.9 ± 7.5 years, 76% female, 89% White). The exposure of interest was all types of antidepressants compared to no use. The mean follow-up was 5 years. The HR was adjusted for age, sex, race and ethnicity, education, comorbidities (e.g., diabetes), smoking, and the presence of APOE e4 allele (i.e., a gene indicating an increased risk of Alzheimer’s and an earlier age of disease onset). The adjusted HR was 0.92 (95% CI = 0.70–1.20) for all antidepressants compared with no use. 

Peakman et al., 2020 [40] was a retrospective cohort study of excellent quality (STROBE-M: 0.90) conducted in the UK. The study population included 3659 older adults with depression and without dementia (mean age: 76.0 ± 7.7 years, 64% female, 82% White). The exposure of interest was all types of antidepressants compared to no use. The mean follow-up was 2.7 years. The HR was adjusted for age, sex, race and ethnicity, marital status, previous diagnosis of depression, comorbidities, and psychotropic use. The adjusted HR was 1.32 (95% CI = 1.01–1.74) for all antidepressants and 1.07 (95% CI = 0.91–1.25) for SSRI compared with no use.

Su et al., 2020 [41] was a retrospective cohort study of excellent quality (STROBE-M: 0.90) conducted in Taiwan. The study population included 138,767 older adults with depression and without dementia (80% aged 60–80 years, 54% female). The exposure of interest was different doses of antidepressant use (cumulative defined daily dosage (cDDD) 28–167 and 168+) compared to no use (defined as cDDD < 28). The grouping approach was based on the empirical prescribing pattern in Taiwan, which was generally less than 4 weeks after the first visit, then 4 weeks and 8 weeks, with a maximum of 12 weeks after the patient reached a stable condition. The mean follow-up was 8 years. The HR was adjusted for age, sex, urbanization, and income. The adjusted HR was 1.06 (95% CI = 0.91–1.23) and 1.07 (95% CI = 0.95–1.20) for the use of lower dose and higher dose, respectively, compared with no use.

### 3.4. Meta-Analysis

**Figure 2:**
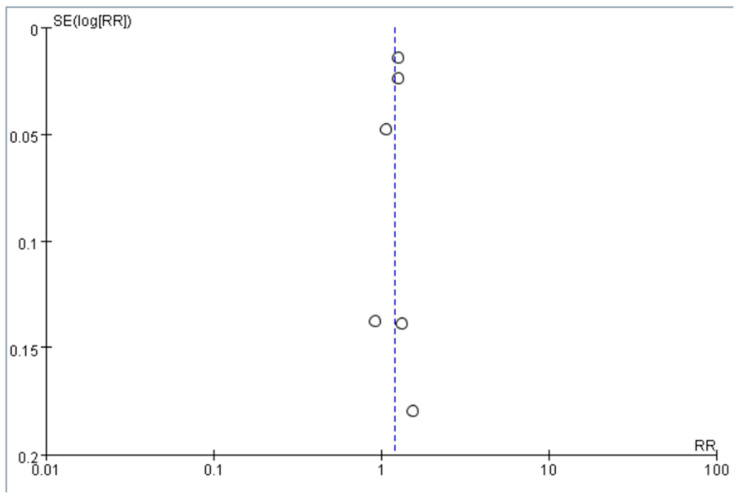
Funnel plot of the included studies. The white circles indicate the RR in each study (RR), and the dashed line indicates the pooled risk ratio across these studies. RR of the included studies are symmetric around this dashed line.

**Figure 3:**
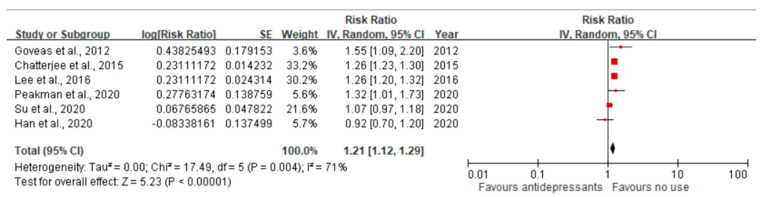
The pooled estimates of the association between antidepressant use and the risk of dementia. The red dot indicates the point estimate of the risk ratio (RR), and the black line indicates the confidence interval (CI). The pooled RR was 1.21 (95% CI 1.12–1.29), indicating that the use of antidepressants was associated with an increased risk of dementia. The heterogeneity across the included studies was moderate to high (I^2^ = 71%) [36,37,38,39,40,41].

We pooled the adjusted RR to quantify the effect of antidepressant use compared to no use on the risk of dementia among older adults with depression. The funnel plot was symmetrical around RR = 1, which did not show significant publication bias (Figure 2). The pooled RR was 1.21 (95% CI = 1.12–1.29), suggesting that compared with no use, the use of antidepressants was associated with an increased risk of dementia among older adults with depression (Figure 3). The heterogeneity across the included studies was moderate to high (I^2^ = 71%). The pooled RR was 1.59 (95% CI = 0.82–3.09) for SSRI and 0.64 (95% CI = 0.09–4.47) for TCA (Figures S1 and S2) compared to no use, which did not show a significant association between SSRI or TCA use and the risk of dementia. However, the implication of the finding was limited due to high heterogeneity (I^2^ = 98% for both SSRI and TCA). 

## 4. Discussion

This meta-analysis found that overall antidepressant use was associated with a higher long-term (≥1-year follow-up) risk of dementia among older adults with depression. This may be explained by the anticholinergic side effect of antidepressants, especially TCAs [16,17]. For example, Chatterjee et al., who followed patients for a median of 3 years, showed that those receiving medications whose anticholinergic drug scale (ranging from 0 to 3) was ≥2 (antidepressants: amitriptyline, clomipramine, doxepin, desipramine, imipramine, nortriptyline, paroxetine, protriptyline, trimipramine) had a 1.26-fold higher odds of dementia compared with those with no use [37]. In addition, mechanistic studies have suggested depression itself may impair cognitive function via several pathophysiological paths, including an elevation of glucocorticoids through hypothalamic–pituitary–adrenal axis dysfunction, an increase in pro-inflammatory cytokines (e.g., tumor necrosis factor-α, interleukin-1β (IL-1β), and IL-6), and deficits in nerve growth factors (e.g., brain-derived neurotrophic factor). These biological mechanisms underlying depression increase the vulnerability of hippocampal atrophy, the deposition of β-amyloid plaques, and tau aggregation, which are the major neuropathological hallmarks of dementia [42,43].

While anticholinergic antidepressants can worsen cognitive functions, some antidepressants might ameliorate cognitive function in depressed patients. For example, Keefe et al. conducted a systematic review of 43 RCTs to examine the effects of SSRIs, SNRIs, TCAs, and other psychotropic agents on cognitive function with a median follow-up duration of 8 weeks [44]. The authors concluded that antidepressants were beneficial in reducing cognitive impairment in patients with major depressive disorder. The immediate benefit of antidepressants on cognitive function is biologically plausible. Several antidepressants have been reported to exert neuroprotective effects through several pathways, including an increase in neurogenesis and reversed neurodegeneration (e.g., fluoxetine, moclobemide, and vortioxetine), an increase in anti-inflammatory cytokines and a reduction in pro-inflammatory markers (e.g., fluoxetine, bupropion, venlafaxine, moclobemide, and vortioxetine), a decrease in oxidative stress (e.g., fluoxetine and vortioxetine), a decrease in β-amyloid plaques (e.g., fluoxetine, citalopram, tranylcypromine, amitriptyline, and vortioxetine), and a decrease in tau aggregation (e.g., escitalopram) [42,45,46,47]. However, our findings suggested that the immediate cognitive benefit may not be strong enough to deter the long-term increased risk of dementia, which could be driven by the progression of depression itself or the use of anticholinergic antidepressants. 

Our study is subject to several limitations. First, the heterogeneity in the main analysis was moderate to high (I^2^ = 71%), probably due to different study locations, study populations, antidepressants included, length of follow-up, and the confounding factors adjusted in each study. We were unable to conduct further stratification or subgroup analysis due to an insufficient number of studies (*n* = 6). Future studies are needed to comprehensively adjust for the severity of depression using the duration or clinical scales (e.g., PHQ-9) to thoroughly account for confounding by indication. However, we restricted the study population to older adults with depression to reduce the selection bias, and the heterogeneity largely decreased compared with a previous meta-analysis that pooled observational studies following older antidepressant users for 3 to 11 years (I^2^ = 99%) [3]. Second, most studies included in this meta-analysis were observational studies, which are susceptible to unmeasured confounding factors (e.g., lifestyle) in the evaluation of causality. However, we pooled the fully adjusted HR/OR to minimize the confounding effect. Most (*n* = 5) studies adjusted for demographic characteristics (e.g., age, sex), comorbidities (e.g., diabetes), and comedications (e.g., statins), while four even adjusted for baseline depressive symptoms and cognitive function. Third, we did not report the differential risks of dementia across all classes of antidepressants due to the lack of information from existing studies. We examined the most commonly used antidepressants in elderly patients, i.e., SSRIs and TCAs, and identified no significantly increased risk of dementia of these therapeutic classes. However, the heterogeneity was very high for both SSRI and TCA (I^2^ = 98%), probably due to the limited number of studies (SSRI: three, TCA: two). Fourth, the cumulative exposure of antidepressants (i.e., dosage and duration) was not evaluated due to insufficient details in the included studies. Fifth, the progress of depression (e.g., severity and duration) may explain the long-term risk of dementia among antidepressant users, but we were not able to obtain such information to control for in the current analyses. Sixth, it is possible that a lack of response to antidepressant therapy leads to a decline in performance and cognitive function. However, patients not receiving antidepressants can also suffer from depression progression and cognitive decline. Seventh, patients (especially non-users) may receive social and psychological support or behavioral treatments including counseling, but the treatments’ effects on risk of dementia were unknown. Eighth, few studies have focused on the association between antidepressant use and specific types of dementia other than Alzheimer’s disease (e.g., frontotemporal dementia, Lewy Body dementia). Despite the limitations, our meta-analysis included the most updated evidence with a follow-up period of at least one year when evaluating the association between antidepressant use and cognitive function. In addition, we focused on patients with depression to increase the homogeneity across the studies. Future studies are needed to account for the severity of depression and to differentiate the effect of depression from that of antidepressants on dementia risk.

## 5. Conclusions

Our results suggest that antidepressant use is associated with an increased risk of dementia in older adults with depression. Future studies are needed to account for the severity of depression and differentiate the effect of depression and antidepressants on dementia risk.

## Figures and Tables

**Table 1:** Summary of the characteristics and findings of the selected articles.

Author, Year	Exposure	Comparison	Follow-Up (Years)	Adjusted RR (95% CI)
Goveas et al., 2012 [36]	ADs	no use	7.6	1.55 (1.09–2.20)
Chatterjee et al., 2015 [37]	Anticholinergic ADs	no use	3	1.26 (1.22–1.29)
Lee et al., 2016 [38]	TCAs	no use	5	0.24 (0.22–0.27)
	SSRIs			2.48 (2.27–2.71)
	MAOIs			1.86 (1.47–2.36)
	Heterocyclic ADs			1.44 (1.32–1.57)
	Other ADs			2.05 (1.85–2.27)
Han et al., 2020 [39]	ADs	no use	5	0.92 (0.70–1.20)
Peakman et al., 2020 [40]	ADs	no use	2.7	1.32 (1.01–1.74)
Su et al., 2020 [41]	ADs	no use: cDDD < 28	8	
	cDDD 28-167			1.06 (0.91–1.23)
	cDDD 168+			1.07 (0.95–1.20)

Abbreviations: ADs, antidepressants; cDDD, cumulative defined daily dosage; MAOI, monoamine oxidase inhibitors; RR, risk ratio; SARI, serotonin antagonist and reuptake inhibitor; SSRIs, selective serotonin reuptake inhibitors; TCA, tricyclic antidepressant.

## Data Availability

No new data were created or analyzed in this study. Data sharing is not applicable to this article.

## Supplementary Materials

The following supporting information can be downloaded at: https://www.mdpi.com/article/10.3390/jcm12196342/s1, Table S1: Preferred Reporting Items for Systematic Reviews and Meta-analyses (PRISMA) 2009 Checklist; Table S2: Search details for each database; Table S3: Characteristics and findings of the selected articles (*n* = 6); Figure S1: The pooled estimates of the association between SSRI use and the risk of dementia; Figure S2: The pooled estimates of the association between TCA use and the risk of dementia.
