# Supplementary material for: Association between Antidepressants and Dementia Risk in Older Adults with Depression: A Systematic Review and Meta-Analysis
Source: J Clin Med. 2023 Oct 3;12(19):6342. doi: 10.3390/jcm12196342 (PMC10573169; doi:10.3390/jcm12196342)
Supplement: Supplementary file 1 [file jcm-12-06342-s001.zip › jcm-2602003-supplementary.pdf]

APPENDIX

Table S1. Preferred Reporting Items for Systematic Reviews and Meta-analyses (PRISMA) 2009 Checklist

Table S2. Search details for each database

Table S3. Characteristics and findings of the selected articles (n=6)

Figure S1. The pooled estimates of the association between SSRI use and the risk of dementia.

Figure S2. The pooled estimates of the association between TCA use and the risk of dementia.

Table S1. Preferred Reporting Items for Systematic Reviews and Meta-analyses (PRISMA) 2009 Checklist

**Section/topic**	#	**Checklist item**	**Reported on page #**
**TITLE**			
**Title**	**1**	Identify the report as a systematic review, meta-analysis, or both.	**1**
**ABSTRACT**			
Structured summary	**2**	Provide a structured summary including, as applicable: background; objectives; data sources; study eligibility criteria, participants, and interventions; study appraisal and synthesis methods; results; limitations; conclusions and implications of key findings; systematic review registration number.	**1**
**INTRODUCTION**			
Rationale	**3**	Describe the rationale for the review in the context of what is already known.	**1-2**
Objectives	**4**	Provide an explicit statement of questions being addressed with reference to participants, interventions, comparisons, outcomes, and study design (PICOS).	**2**
**METHODS**			
Protocol and registration	**5**	Indicate if a review protocol exists, if and where it can be accessed (e.g., Web address), and, if available, provide registration information including registration number.	**1**
Eligibility criteria	**6**	Specify study characteristics (e.g., PICOS, length of follow-up) and report characteristics (e.g., years considered, language, publication status) used as criteria for eligibility, giving rationale.	**2**
Information sources	**7**	Describe all information sources (e.g., databases with dates of coverage, contact with study authors to identify additional studies) in the search and date last searched.	**2**
Search	**8**	Present full electronic search strategy for at least one database, including any limits used, such that it could	**2 & Table S2**

		be repeated.	
Study selection	**9**	State the process for selecting studies (i.e., screening, eligibility, included in systematic review, and, if applicable, included in the meta- analysis).	**2**
Data collection process	**10**	Describe method of data extraction from reports (e.g., piloted forms, independently, in duplicate) and any processes for obtaining and confirming data from investigators.	**3**
Data items	**11**	List and define all variables for which data were sought (e.g., PICOS, funding sources) and any assumptions and simplifications made	**3**
Risk of bias in individual studies	**12**	Describe methods used for assessing risk of bias of individual studies (including specification of whether this was done at the study or outcome level), and how this information is to be used in any data synthesis.	**3**
Summary measures	**13**	State the principal summary measures (e.g., risk ratio, difference in means).	**3**
Synthesis of results	**14**	Describe the methods of handling data and combining results of studies, if done, including measures of consistency (e.g., I2) for each meta-analysis.	**3**
Risk of bias across studies	**15**	Specify any assessment of risk of bias that may affect the cumulative evidence (e.g., publication bias, selective reporting within studies).	**3**
Additional analyses	**16**	Describe methods of additional analyses (e.g., sensitivity or subgroup analyses, meta-regression), if done, indicating which were pre-specified.	**3**
**RESULTS**			
Study selection	**17**	Give numbers of studies screened, assessed for eligibility, and included in the review, with reasons for exclusions at each stage, ideally with a flow diagram.	**3-4**
Study characteristics	**18**	For each study, present characteristics for which data were extracted (e.g., study size, PICOS, follow-up period) and provide the citations.	**4**
Risk of bias within	**19**	Present data on risk of bias of each study and, if	**4**

studies		available, any outcome level assessment (see item 12).	
Results of individual studies	**20**	For all outcomes considered (benefits or harms), present, for each study:<br>(a) simple summary data for each intervention group<br>(b) effect estimates and confidence intervals, ideally with a forest plot.	**5-6**
Synthesis of results	**21**	Present results of each meta-analysis done, including confidence<br>intervals and measures of consistency.	**7**
Risk of bias across studies	**22**	Present results of any assessment of risk of bias across studies (see Item 15).	**7**
Additional analysis	**23**	Give results of additional analyses, if done (e.g., sensitivity or subgroup analyses, meta-regression [see Item 16]).	**7**
**DISCUSSION**			
Summary of evidence	**24**	Summarize the main findings including the strength of evidence for each main outcome; consider their relevance to key groups (e.g., healthcare providers, users, and policy makers).	**8**
Limitations	**25**	Discuss limitations at study and outcome level (e.g., risk of bias), and at review-level (e.g., incomplete retrieval of identified research, reporting bias).	**9**
Conclusions	**26**	Provide a general interpretation of the results in the context of other evidence, and implications for future research.	**9**
**FUNDING**			
Funding	**27**	Describe sources of funding for the systematic review and other support (e.g., supply of data); role of funders for the systematic review.	**9**

Table S2. Search details for each database

Database	#	Search syntax	Citations found
1) Embase	1	(aged OR elderly OR senior OR senium):ti,ab	1246601
	2	(depress* OR melancholia*):ti,ab	691132
	3	(antidepress* OR ((adrenaline OR amine OR monoamine OR monoamino OR mao OR monoaminoxidase) near/3 "oxidase inhibit") OR ((tyraminase OR "tyramine oxidase") near/3 inhibit) OR ((noradrenalin OR noradrenaline OR noradrenaline OR norepinephrine) near/3 "reuptake inhibitor") OR SNRI OR (("serotonin specific" OR "selective serotonin" OR serotonin OR norepinephrine) near/3 "reuptake inhibitor") OR SSRI OR tetracyclic near/3 antidepressant OR tricyclic near/3 antidepressant):ti,ab	115846
	4	"monoamine oxidase inhibitor"/exp OR "noradrenalin uptake inhibitor"/exp OR "serotonin uptake inhibitor"/exp OR "tetracyclic antidepressant agent"/exp OR "tricyclic antidepressant agent"/exp	397631
	5	((cogniti* AND (deficit OR defect OR disability OR disorder OR dysfunction OR impairment OR decline)) OR dementi* OR amnestic OR amentia):ti,ab	415353
	6	"Cognitive defect"/exp OR "Dementia"/exp	554840
	7	#1 AND #2 AND (#3 OR #4) AND (#5 OR #6)	1738
	8	limit #7 to English	1546
2) MEDLINE (PubMed)	1	aged[tiab] OR elderly[tiab] OR senior[tiab] OR senium[tiab]	908604
	2	depress*[tiab] OR melancholia*[tiab]	517974
	3	antidepress*[tiab] OR neurothymoleptic[tiab] OR thymoleptic[tiab] OR thymoanaleptic[tiab] OR psychoenergizer[tiab] OR thymolytic[tiab]	75749

	4	"Antidepressive Agents"[mh] OR Antidepressive Agents [Pharmacological Action]	158967
	5	(cogniti*[tiab] AND (deficit[tiab] OR defect[tiab] OR disability[tiab] OR disorder[tiab] OR dysfunction[tiab] OR impairment[tiab] OR decline[tiab])) OR dementi*[tiab] OR amnestic[tiab] OR amentia[tiab]	291323
	6	"Dementia"[mh] OR "Cognitive Dysfunction"[mh]	205603
	7	#1 AND #2 AND (#3 OR #4) AND (#5 OR #6)	838
	8	limit #7 to English	748
**3) Cochrane CENTRAL**	1	(aged OR elderly OR senior OR senium):ti,ab	164635
	2	(depress* OR melancholia*):ti,ab	86216
	3	(antidepress* OR neurothymoleptic OR thymoleptic OR thymoanaleptic OR psychoenergizer OR thymolytic):ti,ab	14274
	4	[mh "Antidepressive Agents"]	6011
	5	((cogniti* AND (deficit OR defect OR disability OR disorder OR dysfunction OR impairment OR decline)) OR dementi* OR amnestic OR amentia):ti,ab	42048
	6	[mh "Dementia"] OR [mh "Cognitive Dysfunction"]	8201
	7	#1 AND #2 AND (#3 OR #4) AND (#5 OR #6)	305

Table S3. Characteristics and findings of the selected articles (n=6)

Author, year	Study design	Quality	Location	Study population	Mean age (years)	Exposure	Comparison	Outcome	Follow-up (years)	HR/ OR	Adjusted (95% CI)	Adjusted covariates
Goveas et al., 2012[1]	RCT	Low concern	US	Postmenopausal women aged 65–79 years who were with depressive symptoms and without cognitive impairment [a] (n=6,998)	71	ADs (n=383)	no use (n=6,615)	MCI and probable dementia certified by physicians; the etiology of dementia was documented based on the DSM-IV criteria for Alzheimer's disease, vascular dementia, and other dementia-related classifications	7.6	HR	AD: 1.55 (1.09-2.20) SSRI: 1.50 (0.89-2.53) TCA: 1.75 (1.05-2.91)	Demographic characteristics like race, socioeconomic status like education, co-morbidities like diabetes, co-medications like statins, baseline depressive symptoms, physical activity, body mass index, and baseline Modified Mini-Mental State Examination score

Chatterjee et al., 2015[2]	Nested case-control study	Good (0.77)	US	Nursing home older adults with depression and without dementia [b] (n=141,740, 28,388 case and 113,552 control)	80	ADS level 2/3 (ADs: amitriptyline, clomipramine, doxepin, desipramine, imipramine, nortriptyline, paroxetine, protriptyline, trimipramine)	no use	Dementia (all types)	3	OR	1.26 (1.22-1.29)	Demographic characteristics like race, co-morbidities like diabetes, co-medications like statins, and duration of depression
Lee et al., 2016[3]	Case-control study	Excellent (0.92)	Taiwan	Patients aged 40 years or older with major depression and without dementia [b] (n=10,626, 5,394 case and 5,232 control)	75	TCAs, SSRIs, MAOIs, heterocyclic ADs, and other ADs (bupropion, venlafaxine, and mirtazapine)	no use	MCI and dementia (all types)	5	OR	TCA: 0.24 (0.22-0.27) SSRI: 2.48 (2.27-2.71) MAOI: 1.86 (1.47-2.36) hetero: 1.44 (1.32-1.57) other:	Age, sex, other AD use, and comorbidities like diabetes

											2.05 (1.85-2.27)	
Han et al., 2020[4]	Retrospective cohort study	Good (0.82)	US	Older cognitively normal [c] adults with depression (n=716)	72	ADs (n=252)	no use (n=464)	MCI	5	HR	0.92 (0.70-1.20)	Age, sex, race, education, comorbidities like diabetes, smoking, and the presence of the APOE e4 allele
Peakman et al., 2020[5]	Retrospective cohort study	Excellent (0.90)	UK	Older adults with depression and without dementia [b] (n=3,659)	76	ADs (n=3,165)	no use (n=494)	Dementia (all types)	2.7	HR	AD: 1.32 (1.01-1.74) SSRI: 1.07 (0.91-1.25)	Age, sex, ethnicity, marital status, previous diagnosis of depression, comorbidities, psychotropic use
Su et al., 2020[6]	Retrospective cohort study	Excellent (0.90)	Taiwan	Older adults with depression and without dementia [b] (n=138,767)	74	ADs (n=110,687)	no use: cDDD<28 (n=28,080)	Alzheimer's disease certified by physicians	8	HR	cDDD 28-167: 1.06 (0.91-1.23) cDDD 168+:	Gender, age, urbanization, and income

											1.07 (0.95-1.20)	

Abbreviations: AD, antidepressants; ADS, Anticholinergic Drug Scale; cDDD, cumulative defined daily dosage; CI, confidence interval; HR, hazard ratio; MAOI, Monoamine Oxidase Inhibitors; MCI, mild cognitive impairment; OR, odds ratio; RCT, randomized controlled trial; SARI, Serotonin Antagonist and Reuptake Inhibitor; SSRIs, Selective Serotonin Reuptake Inhibitors; TCA, Tricyclic Antidepressant

a: This was ascertained by the Modified Mini-Mental State Examination.

b: This was ascertained by the diagnostic code.

c: This was ascertained by either a single clinician or a formal consensus panel.

Study or Subgroup	log[Risk Ratio]	SE	Weight	Risk Ratio IV, Random, 95% CI	Year
Goveas et al., 2012	0.40546511	0.266519	29.3%	1.50 [0.89, 2.53]	2012
Lee et al., 2016	0.90825856	0.045196	35.6%	2.48 [2.27, 2.71]	2016
Peakman et al., 2020	0.06765865	0.080983	35.1%	1.07 [0.91, 1.25]	2020
**Total (95% CI)**			**100.0%**	**1.59 [0.82, 3.09]**	

Heterogeneity: Tau² = 0.32; Chi² = 83.42, df = 2 (P < 0.00001); I² = 98%
Test for overall effect: Z = 1.38 (P = 0.17)

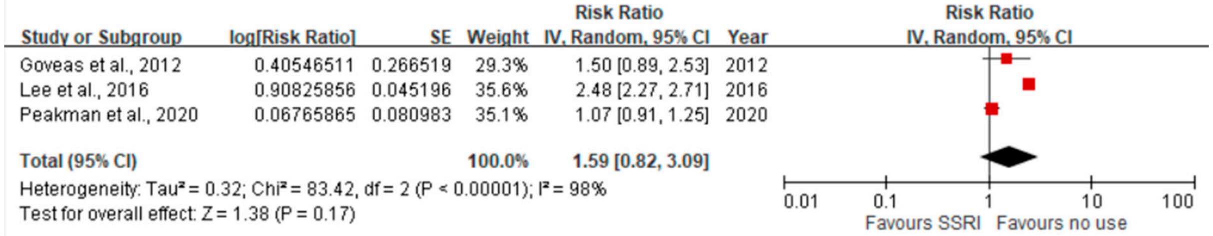

Figure S1. The pooled estimates of the association between SSRI use and the risk of dementia.

Study or Subgroup	log[Risk Ratio]	SE	Weight	Risk Ratio IV, Random, 95% CI	Year
Goveas et al., 2012	-1.42711636	0.052243	50.8%	0.24 [0.22, 0.27]	2012
Lee et al., 2016	0.55961579	0.260042	49.2%	1.75 [1.05, 2.91]	2016
**Total (95% CI)**			**100.0%**	**0.64 [0.09, 4.47]**	

Heterogeneity: Tau² = 1.94; Chi² = 56.11, df = 1 (P < 0.00001); I² = 98%
Test for overall effect: Z = 0.45 (P = 0.65)

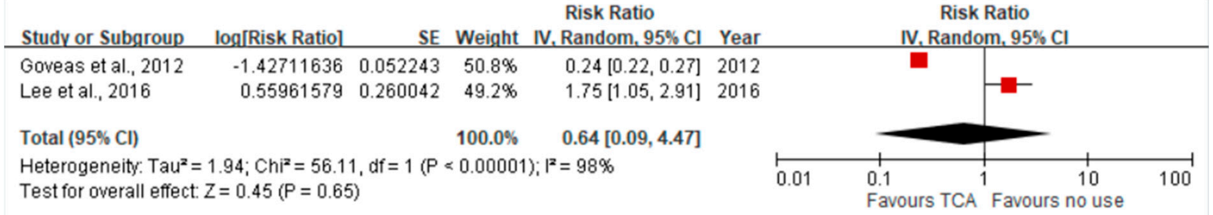

Figure S2. The pooled estimates of the association between TCA use and the risk of dementia.

References

1. Goveas, J.S.; Hogan, P.E.; Kotchen, J.M.; Smoller, J.W.; Denburg, N.L.; Manson, J.E.; Tummala, A.; Mysiw, W.J.; Ockene, J.K.; Woods, N.F. Depressive symptoms, antidepressant use, and future cognitive health in postmenopausal women: the Women's Health Initiative Memory Study. *International psychogeriatrics* **2012**, *24*, 1252-1264.
2. Chatterjee, S.; Bali, V.; Carnahan, R.M.; Johnson, M.L.; Chen, H.; Aparasu, R.R. Anticholinergic medication use and risk of dementia among elderly nursing home residents with depression. *The American Journal of Geriatric Psychiatry* **2016**, *24*, 485-495.
3. Lee, C.W.-S.; Lin, C.-L.; Sung, F.-C.; Liang, J.-A.; Kao, C.-H. Antidepressant treatment and risk of dementia: a population-based, retrospective case-control study. *The Journal of clinical psychiatry* **2016**, *77*, 961.
4. Han, F.; Bonnett, T.; Brenowitz, W.D.; Teylan, M.A.; Besser, L.M.; Chen, Y.-C.; Chan, G.; Cao, K.-G.; Gao, Y.; Zhou, X.-H. Estimating associations between antidepressant use and incident mild cognitive impairment in older adults with depression. *PloS one* **2020**, *15*, e0227924.
5. Peakman, G.; Karunatilake, N.; Seynaeve, M.; Perera, G.; Aarsland, D.; Stewart, R.; Mueller, C. Clinical factors associated with progression to dementia in people with late-life depression: A cohort study of patients in secondary care. *BMJ open* **2020**, *10*, e035147.
6. Su, J.-A.; Chang, C.-C.; Yang, Y.-H.; Chen, K.-J.; Li, Y.-P.; Lin, C.-Y. Risk of incident dementia in late-life depression treated with antidepressants: A nationwide population cohort study. *Journal of Psychopharmacology* **2020**, *34*, 1134-1142.
